# Thyroid cancer: trends in incidence, mortality and clinical-pathological patterns in Zhejiang Province, Southeast China

**DOI:** 10.1186/s12885-018-4081-7

**Published:** 2018-03-15

**Authors:** Lingbin Du, Youqing Wang, Xiaohui Sun, Huizhang Li, Xinwei Geng, Minghua Ge, Yimin Zhu

**Affiliations:** 1 Zhejiang Cancer Center, Zhejiang Cancer Hospital, No.30 Jichang Road, Hangzhou Zhejiang, 310004 China; 2 Department of Epidemiology & Biostatistics, Zhejiang University School of Public Health, 388 Yu-Hang-Tang Road, Zhejiang, Hangzhou 310058 China; 3 Department of Pharmacology, Zhejiang University School of Medicine, Hangzhou, 310058 China; 4 Head and Neck Surgery, Zhejiang Cancer Hospital, No.1 East Banshan Road, Hangzhou, Zhejiang 310022 China

**Keywords:** Thyroid cancer, PTMC, Incidence, Mortality, Pathological classifications, Over diagnosis

## Abstract

**Background:**

Thyroid cancer is the most common malignant disease of the endocrine system. Previous studies indicate a rapid increase in the incidence of thyroid cancer in recent decades, and this increase has aroused the great public concern. The aim of this study was to analyze the trends in incidence, mortality and clinical-pathological patterns of thyroid cancer in Zhejiang province.

**Methods:**

Population-based incidence and mortality rates of thyroid cancer were collected from eight cancer registries in Zhejiang from 2000 to 2012. The incidence and mortality rates were age-standardized to Segi’s world population. A Joinpoint model was used to examine secular trends in age-adjusted thyroid cancer rates with the Joinpoint Regression Program Version 4.0.0. Thyroid cancer patients were recruited from Zhejiang Cancer Hospital from 1972 to 2014. Patient demographics, tumor histology and tumor size were compared among the different periods of 1972–1985, 1986–1999 and 2000–2014.

**Results:**

The age-standardized incidence rate of thyroid cancer in Zhejiang cancer registries was 2.75/10^5^ in 2000, and increased to 19.42/10^5^ in 2012. Additionally, we observed significantly increasing incidence rates with the Annual Percent Change (APC) of 22.86% (95%CI, 19.2%–26.7%). The age-standardized mortality of thyroid cancer in Zhejiang cancer registries was 0.23/10^5^ in 2000 and 0.25/10^5^ in 2012. No significant change in mortality rate was found. We observed a rapid increase in the proportions of papillary thyroid carcinoma (PTC) in 12,508 patients with thyroid carcinoma identified in the Zhejiang Cancer Hospital from 1972 to 2014 while the proportions of poorly differentiated thyroid cancer (PDTC), medullary thyroid carcinoma (MTC) and follicular thyroid carcinoma (FTC) decreased over the decades. In the PTC cases, the proportion of patients with maximum tumor diameter (MTD) < 1 cm dramatically and significantly increased from 0 in 1972–1985 to 32.1% in 2000–2014.

**Conclusions:**

A rapid increase in incidence and a stable trend in mortality of thyroid cancer were found in the distribution of thyroid cancer. Most of the increased incidence was PTC, especially the papillary thyroid microcarcinoma (PTMC) with MTD < 1 cm. This increase in incidence might be due to increased diagnosis with advanced technology.

## Background

Thyroid cancer is a relatively rare neoplasm worldwide [1], but the most common malignant disease of the endocrine system [2]. A rapid increase in the incidence rate of thyroid cancer has been reported in the past several decades in many countries including China [3–6]. The global age-standardized thyroid cancer incidence rates had an average increase of 58.1% from 1970 to 2002 [3], with geographical differences. Northern European countries, Australia and Japan experienced a relatively low incidence and a little increase [7, 8] whereas steep upward trends of thyroid cancer were observed in southern European countries [9, 10], the United States [11–13] and most markedly in the Republic of Korea [14]. In China, a clear increase in the incidence was also reported by previous studies [15–17]. The histologic type of thyroid cancer which contributes to the highest increase in the incidence in the world is papillary thyroid cancer (PTC) [10, 12, 18]. This rapid increase has caused widespread public concern about thyroid cancer.

The underlying causes of this increasing trend remain unknown. This change may be partly attributable to environmental risk factors, including a deficit or excess of iodine intake [19], medical radiation [20] and nutrition-related factors [21]. The use of advanced diagnostic techniques, such as ultrasound examination, computed tomography and magnetic resonance imaging scanning, and much more sensitive biochemical markers, which are more prone to the discovery of the thyroid nodules, have been proposed as the main reasons for the increasing trend [14, 22–24].

On the other hand, quite a few studies have reported that the mortality rates have not increased or have even declined in most countries in the world [7, 25, 26]. Therefore, the following questions were raised: (1) What is the reason for different trends between incidence and mortality of thyroid cancer? (2) What is the changing trend in clinical-pathological parameters? To address these questions, we analyzed the temporal distributions of thyroid cancer incidence and mortality in the population-based cancer registries of Zhejiang Province from 2000 to 2012, and the trends in the clinical-pathological parameters of thyroid cancer patients in Zhejiang Cancer Hospital from 1972 to 2014.

## Methods

### Data source

Incidence and mortality data of thyroid cancer from 2000 to 2012 were obtained from eight cancer registries (Jiashan, Jiaxing City, Haining, Hangzhou city, Shangyu, Xianju, Kaihua and Cixi). In 2012, these eight cancer registries covered a population of 11,127,744 and accounting for 20.34% of the total population in Zhejiang Province. The cancer incidence and mortality data were collected from each cancer registry and then aggregated in Zhejiang Cancer Center (ZJCC). ZJCC is responsible for evaluation and analysis of cancer data. Thyroid cancer cases were identified per the International Classification of Diseases for Oncology, 3rd edition (ICD-O-3) and the International Statistical Classification of Diseases and Related Health Problems 10th Revision (ICD-10).

The clinical-pathological data for thyroid cancer patients from 1972 to 2014 were collected from inpatients in Zhejiang Cancer Hospital, a leading regional cancer hospital in Zhejiang Province. The cancer patients in this hospital represent approximately 30% of all the cancer patients in Zhejiang province. The classification of thyroid cancer was based on ICD-8, ICD-9, and ICD-10 for the period 1972–1988, 1989–1995, and 1996–2001, respectively. There were no changes in the coding of thyroid cancer among the 8th, 9th, and 10th revisions [3]. During the period 2002–2014, ICD-O was used for coding morphology (2nd edition, for the period 2002–2003 and 3rd edition for later period). All patients with thyroid cancer were identified and the reports from the clinicians or pathologists were examined for every case to establish the histological type. Thyroid cancer was classified into 4 groups based on the criteria of WHO [27]: PTC, MTC, FTC, and PDTC (including anaplastic and other/unspecified).

### Statistical analysis

The incidence and mortality rates were age-standardized using Segi’s world population. Temporal trends of thyroid cancer incidence and mortality rates from 2000 to 2012 were evaluated with annual percentage change (APC) using the Joinpoint Regression Program Version 4.0.0 (Statistical Research and Applications Branch, National Cancer Institute). The Joinpoint model evaluates changing linear trends across consecutive time periods [28]. Distribution differences in the demographics, tumor histology and tumor size were compared among three periods of 1972–1985, 1986–1999 and 2000–2014. Numerical data were presented as mean ± standard deviation (SD), and enumeration data were presented as frequency (%). Data were analyzed using the Student’s *t* test and Kruskal-Wallis *H* test for continuous variables, and the *χ*^*2*^ test for categorical variables. A *P* value < 0.05 was considered to be statistically significant. All hypothesis tests were two sided. Statistical analyses were performed using SPSS 22.0 (SPSS, Chicago, IL, USA).

## Results

### Rapidly increasing trends in thyroid cancer incidence from 2000 to 2012

During the period 2000–2012, the eight cancer registries covered a total population of 92,886,021 person-years (73,680,781 in urban and 19,205,240 in rural areas), including 41,315,911 person-years for males and 40,442,366 person-years for females.

**Fig. 1:**
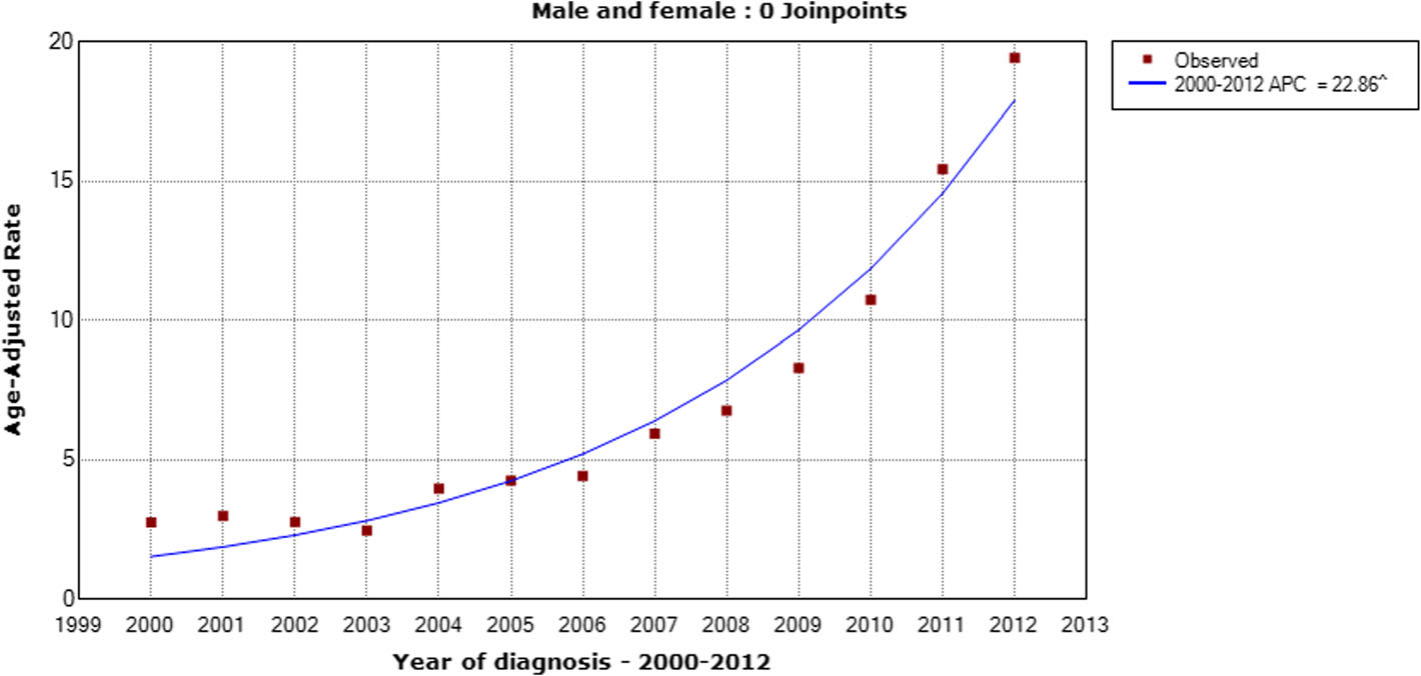
The Joinpoint analysis of age-standardized incidence rates for thyroid cancer in Zhejiang, 2000–2012

**Fig. 2:**
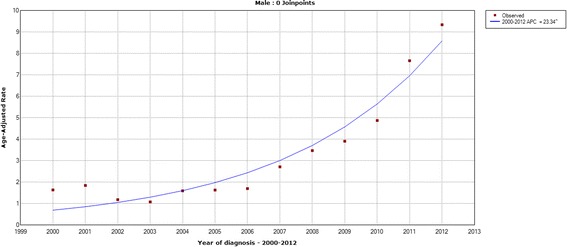
The Joinpoint analysis of age-standardized incidence rates in males for thyroid cancer in Zhejiang, 2000–2012

**Fig. 3:**
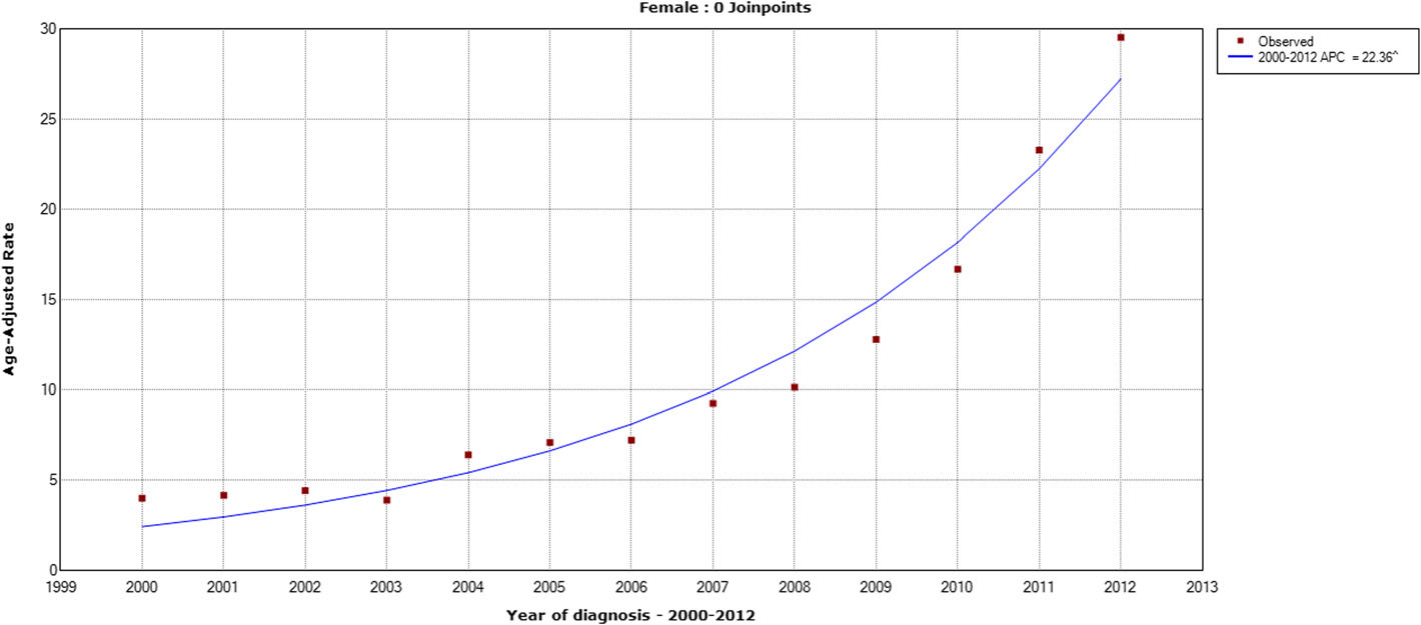
The Joinpoint analysis of age-standardized incidence rates in females for thyroid cancer in Zhejiang, 2000–2012

**Fig. 4:**
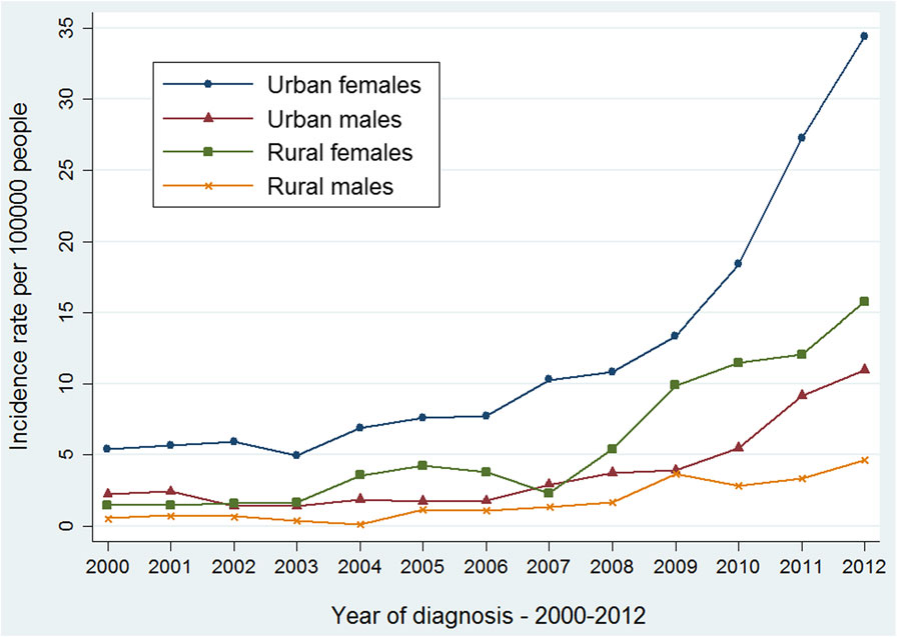
Age-standardized (per 100,000 world standard) incidence rates of thyroid cancer in Zhejiang, 2000–2012

The age-standardized incidence rates of thyroid cancer in Zhejiang cancer registries from 2000 to 2012 are shown from Figs. 1, 2, 3 and 4. The overall age-standardized incidence rate was 2.75/10^5^ in 2000 and 19.42/10^5^ in 2012. Compared with the incidence rate in 2000, the rate in 2012 increased by 6.06 times. The Joinpoint analysis revealed an APC of 22.86% (95% CI, 19.2%–26.7%) (Fig. 1). However, different changing patterns were found in different periods. The APC was 0.06% in the period 2000–2003, 14.02% in the period 2003–2006, and 29.59% in the period 2006–2012, respectively. Similar increasing trends were also observed in males (Fig. 2) and females (Fig. 3). We concluded that the incidence rates of females were higher than those of males, and the incidence rates in urban areas were higher than those in rural areas (Fig. 4). These figures showed that the incidence increased rapidly in the past decade.

### Stable trends in thyroid cancer mortality from 2000 to 2012

**Fig. 5:**
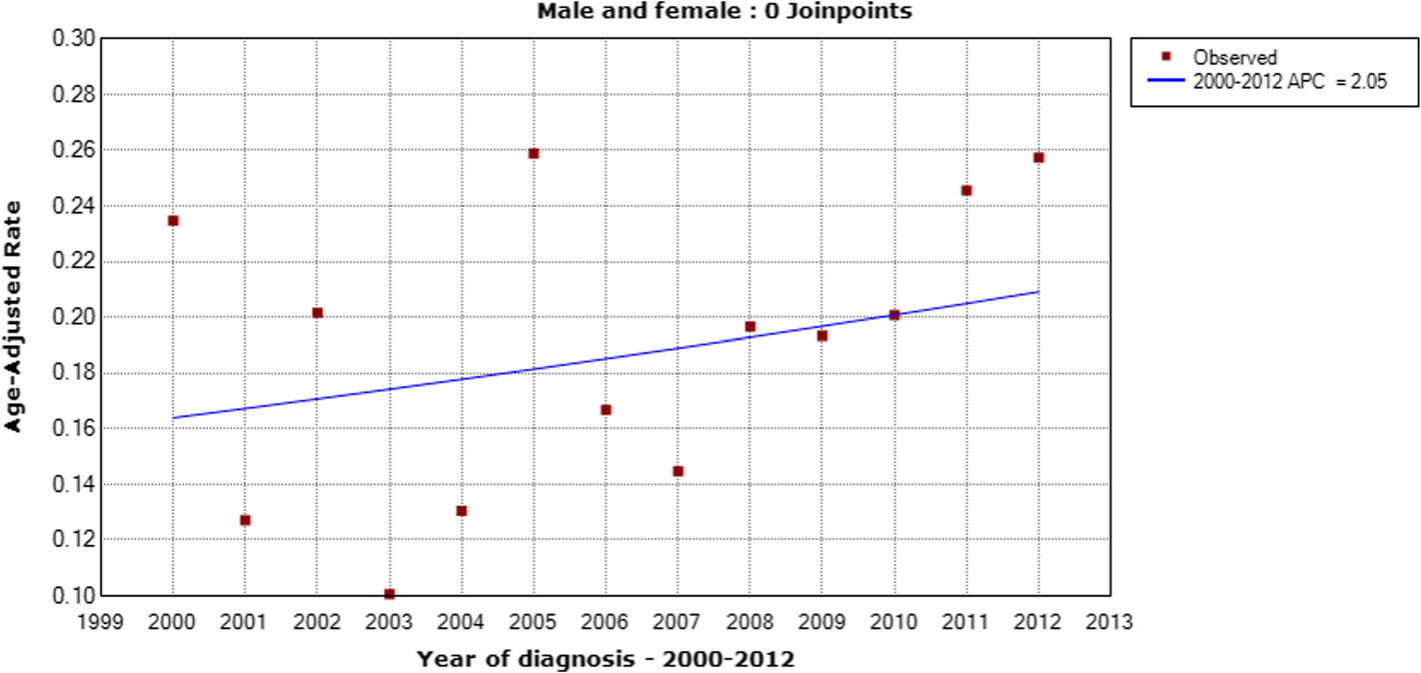
The Joinpoint analysis of age-standardized mortality rates for thyroid cancer in Zhejiang, 2000–2012

**Fig. 6:**
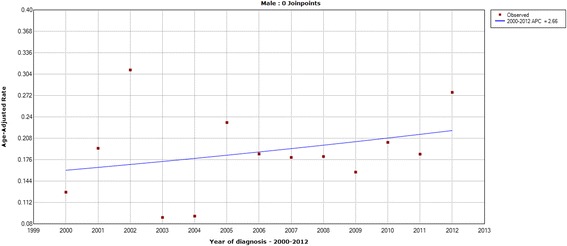
The Joinpoint analysis of age-standardized mortality rates in males for thyroid cancer in Zhejiang, 2000–2012

**Fig. 7:**
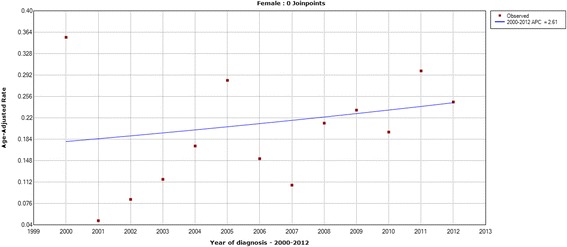
The Joinpoint analysis of age-standardized mortality rates in females for thyroid cancer in Zhejiang, 2000–2012

**Fig. 8:**
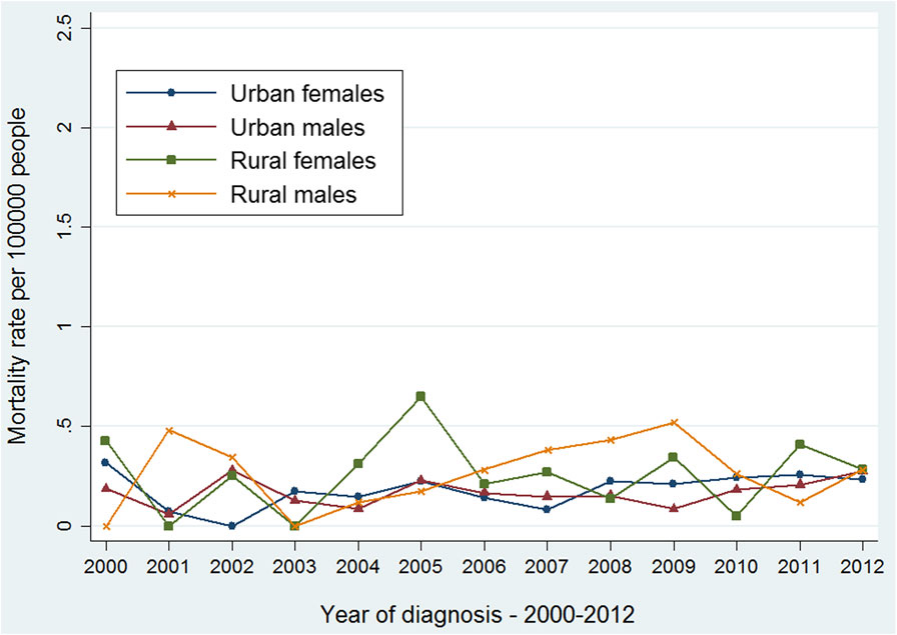
Age-standardized (per 100,000 world standard) mortality rates of thyroid cancer in Zhejiang, 2000–2012

The age-standardized mortality rates of thyroid cancer are described from Figs. 5, 6, 7 and 8. The age-standardized mortality rate was 0.23/10^5^ in 2000 and 0.25/10^5^ in 2012. The mean mortality rate during this period was 0.20/10^5^, and the minimum and maximum of the rates were 0.10/10^5^ and 0.26/10^5^, respectively. The Joinpoint analysis revealed no statistically significant difference with an APC of 2.05% (95% CI, − 1.7%-6.0%) (Fig. 5). Similar patterns of mortality trend were found in males (Fig. 6) and females (Fig. 7), and in urban and rural areas (Fig. 8), respectively. These results showed that the mortality of thyroid cancer remained stable in the past decade.

### Trends in age of onset, sex and pathological classification of patients from 1972 to 2014

A total of 12,508 patients with thyroid carcinoma were recruited in Zhejiang Cancer Hospital from 1972 through 2014. We classified the period 1972–2014 into three groups including 1972–1985, 1986–1999 and 2000–2014. Table 1 shows the changing trends of age of onset, sex distribution and pathological classifications of thyroid cancer in different periods. The mean age of onset was 44.7 years old. The age of onset has gradually and significantly increased over time, from a mean age of 39.9 years (SD =13.9 years) for the period 1972–1985 to a mean age of 45.1 (SD =12.3 years) for the period 2000–2014 (*P* < 0.001). The mean age of onset in female patients (44.3 ± 12.2) was significantly lower than those in males (45.8 ± 13.3). Over the past decades, there has been a higher and higher percentage of females compared to males (*P* < 0.001). The overall male-to-female sex ratio of thyroid cancer was 0.38.

**Table 1:** Changing trends of onset age, sex distribution and pathological classifications of thyroid cancer in different periods

	Overall	1972–1985	1986–1999	2000–2014	*P*
Age at diagnosis					
Overall	44.7 ± 12.5	39.9 ± 13.9	41.3 ± 13.8	45.1 ± 12.3	< 0.001
Male	45.8 ± 13.3	41.7 ± 15.7	45.9 ± 14.6	45.9 ± 13.1	0.076
Female	44.3 ± 12.2	38.4 ± 12.1	39.3 ± 13.0	44.8 ± 12.0	< 0.001
*P*	< 0.001	0.209	< 0.001	< 0.001	
Sex					
Male	3458 (27.6)	54 (45.4)	334 (30.3)	3070 (27.2)	< 0.001
Female	9050 (72.4)	65 (54.6)	768 (69.7)	8217 (72.8)	
Male-to-female ratio	0.38	0.83	0.43	0.37	
Pathological classifications					
PTC^a^	10,979 (94.9)	73 (78.5)	795 (82.9)	10,111 (96.1)	< 0.001
FTC^b^	303 (2.6)	11 (11.8)	65 (6.8)	227 (2.2)	
MTC^c^	214 (1.8)	5 (5.4)	72 (7.5)	137 (1.3)	
PDTC^d^	73 (0.6)	4 (4.3)	27 (2.8)	42 (0.4)	

Ages are presented as mean years ± SD and others are presented as frequency (%). The sum of the numbers for some characteristic variables is less than the total due to missing values

^a^PTC represents papillary thyroid carcinoma

^b^FTC represents follicular thyroid carcinoma

^c^MTC represents medullary thyroid carcinoma

^d^PDTC represents poorly differentiated thyroid cancer

The pathological morphology of thyroid cancer was divided into four groups including PTC, MTC, FTC, and PDTC. During the period 1972–1985, 78.5% of thyroid cancer were PTC, 11.8% were FTC, 5.4% were MTC, and 4.3% were PDTC. However, during the period 2000–2014, 96.1% were PTC, 2.2% were FTC, 1.3% were MTC, and only 0.4% were PDTC. A statistical significant difference was found in the distribution of pathological classification (*P* < 0.001). The proportions of PDTC and FTC decreased while the proportion of PTC rapidly increased over the decades.

Table 2 presents the distribution of MTD of the PTC by sex, age and period. The total number of PTC patients for the period 1972–2014 was 10,979. The PTC patients were divided into two groups with MTD < 1 cm and > 1 cm. The percentage of patients with MTD < 1 cm in females was 30.7%, which was slightly and significantly higher than that in males. Moreover, the highest proportion of PTC cases was in the age group of 40–59 years. In PTC cases, the proportion of the tumors with MTD < 1 cm was none in 1972–1985. However, in 2000–2014, this proportion dramatically increased to 32.1%. A significant increase in the trend of the proportion of the tumors with MTD < 1 cm was found from 1972 to 2014 (*P* < 0.001).

**Table 2:** Distribution of maximum tumor diameter (MTD) of the papillary thyroid carcinoma (PTC) by sex, age and period

	MTD < 1 cm	MTD > 1 cm	*P*
Overall	3245 (29.6)	7734 (70.4)	
Sex			
Male	762 (26.2)	2142 (73.8)	< 0.001
Female	2483 (30.7)	5592 (69.3)	
Age (year)			
< 20	7 (4.1)	162 (95.9)	< 0.001
20-	887 (23.9)	2826 (76.1)	
40-	2046 (34.3)	3913 (65.7)	
60-	305 (26.9)	830 (73.1)	
Period			
1972–1985	0	73 (100.0)	< 0.001
1986–1999	3 (0.4)	792 (99.6)	
2000–2014	3242 (32.1)	6869 (65.3)	

Data are presented as frequency (%). The sum of the numbers for some characteristic variables is less than the total due to missing values

**Fig. 9:**
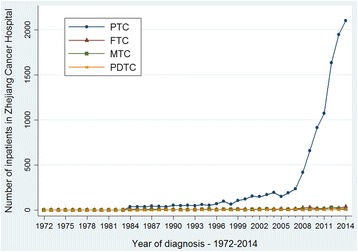
The temporal changes of the number of different histological subtypes of thyroid cancer in Zhejiang Cancer Hospital, 1972–2014

**Fig. 10:**
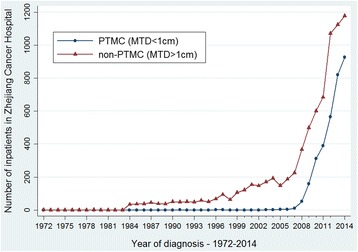
The temporal changes of the number of different sizes of PTC cases in Zhejiang Cancer Hospital, 1972–2014

Figure 9 shows the temporal changes of the number of different histological subtypes of thyroid cancer in Zhejiang Cancer Hospital from 1972 to 2014. We can clearly see that PTC cases increased dramatically with time, while the other three subtypes remained stable. Figure 10 shows the temporal changes of the number of different sizes of PTC cases. We can see that the PTMC cases were rare before 2008 and increased sharply since 2008.

## Discussion

In our study, we found an ongoing epidemic of thyroid cancer in Zhejiang, China. The incidence of thyroid cancer increased remarkably from 2000 to 2012 with an APC of 22.86%. During the period 2000–2012, the incidence rates dramatically changed more than 7-fold, while the mortality rates remained stable. Furthermore, from 1972 to 2014, we observed an increase in the proportion of PTC occurring in Zhejiang Cancer Hospital, especially papillary thyroid microcarcinoma (PTMC) with MTD < 1 cm.

Thyroid cancer accounts for only 1–2% of all malignancies worldwide [29], but despite this, it remains the most common endocrine malignancy [30]. In 2012, the global age-standardized incidence rate by world population (ASIRW) of thyroid cancer was 4.0/10^5^, while China had at a relatively low level of incidence (the ASIRW was 2.8/10^5^) [31]. Zhejiang is an eastern coastal province of China, which belongs to comparatively economically developed regions [15]. In a previous report, we concluded that the ASIRW of thyroid cancer in Zhejiang was obviously much higher than the national level. For example, in the year of 2010, the ASIRW of thyroid cancer in Zhejiang was 10.74/10^5^, and the ASIRW of thyroid cancer throughout China was only 3.23/10^5^ by contrast [31]. Thus, more attention should be focused on thyroid cancer prevention and control in Zhejiang. Compared with our previous study [32] published in 2014, this current study with more information and larger sampling size places emphasis on the secular trends of incidence and mortality, while the previous study provided a more comprehensive description of the epidemiological characteristics of thyroid cancer in six cancer registries of Zhejiang during 2000–2009. By comparison, the APC of incidence from 2000 to 2009 was 16.32% [32], while the APC in 2000–2012 was 22.86%; furthermore, this current study showed that the APC was 0.06% in 2000–2003, 14.02% in 2003–2006, and 29.59% in 2006–2012. The APC of mortality from 2000 to 2009 was 2.14% [32], while the APC from 2000 to 2012 decreased to 2.05%. Thus, we concluded that the incidence of thyroid cancer in Zhejiang has increased rapidly in recent years, whereas the mortality has remained relatively stable or even decreased.

The variation in the incidences of thyroid cancer during recent decades is mainly attributable to PTC. PTC is the most common type of thyroid cancer, with a low degree of malignancy and a good prognosis [1]. Moreover, our findings suggest that the diagnosis of PTMC has contributed to the dramatic increase in cases since 2008. And in Zhejiang Province, the B ultrasonography of thyroid was gradually included in the routine physical examination starting from the years of 2008–2010, and now has been spread to the whole province. Because of the use of B ultrasonography, abnormalities often present in people who hardly develop symptoms [33, 34]. This argument is corroborated by other epidemiological studies showing an increase in PTC diagnosis at the expense of PTMC [11, 35]. These tumors are known to have a lower risk and better surgical outcome. Davies and Welch [11] found that the major increases in PTC were in tumors < 2 cm in USA from 1988 to 2002. Using a cohort of 13,793 thyroidectomies patients performed over 40 years, Grodski et al. concluded that there was no increase in cancer incidence when PTMC were excluded [36].

There seems to be an over diagnosis epidemic instead of an epidemic disease. Researchers concluded in the past that the increase in the incidence rates reflects the increased detection of subclinical disease, not an increase in the true occurrence of thyroid cancer due to the increase in PTMC [11]. Davies L et al. found that the incidence of thyroid cancer in the United States more than doubled over the past 30 years and that 87% of the increase was due to the diagnosis of small papillary cancers [11]. Our study also showed that the incidence of non-PTMC (papillary thyroid carcinoma with MTD > 1 cm) increased during the last 42 years. However, the change in trend of PTMC was more obvious than that of non-PTMC. Most probably, the trends reflect an increase in the environmental risk, boosted by augmented diagnostic activity, following more careful pathological examination. The presumed explanation for the observed increasing trend of thyroid cancer is the advanced diagnostic technique or increased attention to small nodules.

Data from Zhejiang Cancer Hospital showed that the highest proportion of the thyroid cancer cases was in the 40–59 age group. A possible explanation is that the residents aged 40–59 years might pay more attention to the physical examinations as they enter early old age. Moreover, compared to rural areas, papillary thyroid cancer incidence rates started higher in urban areas and increased faster. The detection level in urban areas is greater than that in rural areas because the urban residents seem to take the physical examination far more seriously. A Chinese study has demonstrated that more than 80% of the thyroid cancer cases in Hangzhou, the capital city in Zhejiang Province, were detected due to routine physical examinations [32].

It is currently unclear whether the observed increase in thyroid cancer is real or due to increased diagnosis. However, it has been shown that the increase of comprehensive medical tests in the general population has led to an increased diagnosis of thyroid cancer; the impacts of changes in medical practices and the refined diagnostic techniques have been reported by many authors [3, 37, 38]. Diagnostic sensitivity and opportunities for detection have improved over the past decades with the introduction of thyroid ultrasound in the early 1980s and final-needle aspiration technology in the late 1980s [2]. These technologies could have potentially impacted the secular trends in one gender more than the other. We found statistically significant temporal and age-related differences for females and males. The incidence of thyroid cancer is 2.26–4.35 folds higher in females than in males. This is particularly notable partly because women’s oestrogen level is proved to be one of the risk factors of thyroid cancer, and the oestrogen level is actually higher in women than it is in men [3]. This finding is similar to that of Louise Davies’ study [12]. A recent study claimed that diagnostic changes may account for ≥ 60% of thyroid cancer cases diagnosed in women aged under 80 in many countries and approximately 50% in other countries, except Japan (30%) [22]. A potential explanation for the rapid increase in incidence observed among females occurring early in life may be greater detection during annual obstetrical and gynecological examinations during the reproductive years, whereas the slower rise in incidence among males might reflect more frequent medical visits later in life.

In addition, there is concern about the increase in background irradiation that has occurred over the past decades [39]. It is possible that increasing exposure to radiation from greater use of diagnostic imaging may be contributing to the increasing incidence of thyroid cancer [40]. The iodine excess in the diet might also lead to the increasing incidence of PTC, while iodine levels in Zhejiang Province are considered to be sufficient [32]. Some researchers presented the viewpoints that familial inheritance, mental factors and obesity could be related to the incidence of thyroid cancer [3].

However, this study has limitations. Firstly, the population-based cancer registries did not have enough details in clinical-pathological characteristics including tumor type and size. So the clinical-pathological data were obtained from Zhejiang Cancer Hospital. However, this hospital has approximately 30% of all the cancer patients in Zhejiang province and these proportions remained stable in the study period. Therefore, the trend of clinical parameters in this hospital reflects the overall trend of cancer patients in Zhejiang province. Secondly, it does not provide the exact explanations for how the patients get increased diagnosed. We assume from our study that the potential reason is the individual’s exposure to medical care. The higher incidence of thyroid cancer in residents may be due to the medical tests excluding the other risk factors. Thirdly, since the diagnostic tool, histological criteria, and ICD coding had been modified significantly over a long period (1972–2014), the reclassification might be relatively difficult and the accuracy of these analyses might be lowered.

## Conclusion

Based on the data of population-based surveillance data and clinical pathology, we found rapidly increasing incidence and stable mortality of thyroid cancer. The increased cases classified primarily as a low-risk subtype of PTC, with favorable prognosis. One presumed explanation for this increase of thyroid cancer is increased diagnosis. Our findings suggest that increased incidence reflects increased detection of subclinical tumors, not a real ongoing epidemic of thyroid cancer. Further studies are needed to explore the real reason for the increasing incidence of thyroid cancer.

## Abbreviations

APC: Annual percentage change
ASIRW: Age-standardized incidence rate by world population
FTC: Follicular thyroid carcinoma
ICD: The International Statistical Classification of Diseases and Related Health Problems
ICD-O: The International Classification of Diseases for Oncology
MTC: Medullary thyroid carcinoma
MTD: Maximum tumor diameter
PDTC: Poorly differentiated thyroid cancer
PTC: Papillary thyroid carcinoma
PTMC: Papillary thyroid microcarcinoma
SD: Standard deviation
